# (2,2′-Bipyridyl-κ^2^ *N*,*N*′)bis­(*N*-butyl-*N*-methyl­dithio­carbamato-κ^2^ *S*,*S*′)cadmium(II)

**DOI:** 10.1107/S1600536811006878

**Published:** 2011-02-26

**Authors:** Nur Amirah Jamaluddin, Ibrahim Baba, Mohamed Ibrahim Mohamed Tahir, Edward R. T. Tiekink

**Affiliations:** a School of Chemical Sciences and Food Technology, Faculty of Science and Technology, Universiti Kebangsaan Malaysia, 43600 Bangi, Malaysia; b Department of Chemistry, Universiti Putra Malaysia, 43400 Serdang, Malaysia; c Department of Chemistry, University of Malaya, 50603 Kuala Lumpur, Malaysia

## Abstract

The Cd^II^ atom in the title compound, [Cd(C_6_H_12_NS_2_)_2_(C_10_H_8_N_2_)], is hexa­coordinated by two dithio­carbamate ligands and the N atoms from a bidentate 2,2′-bipyridyl mol­ecule. The coordination geometry is based on a distorted trigonal–prismatic arrangement of the N_2_S_4_ donor set. Supra­molecular chains, aligned along the *a*-axis direction, are mediated by C—H⋯S inter­actions and these are connected into layers that stack along the *c* axis *via* π–π inter­actions [*Cg*(pyrid­yl)⋯*Cg*(pyrid­yl) = 3.6587 (13) Å].

## Related literature

For background to supra­molecular polymers of zinc-triad 1,1-dithiol­ates, including dithio­carbamates, see: Chen *et al.* (2006 ▶); Benson *et al.* (2007 ▶). For a closely related 2,2′-bipyridyl adduct, see: Song & Tiekink (2009 ▶). 
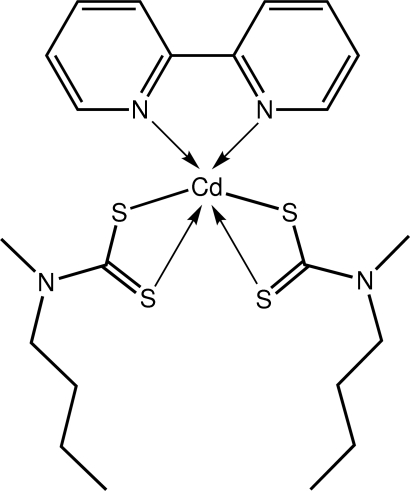


## Experimental

### 

#### Crystal data

[Cd(C_6_H_12_NS_2_)_2_(C_10_H_8_N_2_)]
*M* *_r_* = 593.16
Triclinic, 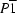

*a* = 10.3215 (4) Å
*b* = 10.6465 (4) Å
*c* = 12.4546 (5) Å
α = 81.566 (3)°
β = 74.790 (3)°
γ = 83.641 (3)°
*V* = 1302.64 (9) Å^3^
*Z* = 2
Mo *K*α radiation
μ = 1.18 mm^−1^
*T* = 150 K
0.27 × 0.16 × 0.01 mm

#### Data collection

Oxford Diffraction Xcaliber Eos Gemini diffractometer
Absorption correction: multi-scan (*CrysAlis PRO*; Oxford Diffraction, 2010 ▶) *T*_min_ = 0.829, *T*_max_ = 0.990
15837 measured reflections
5393 independent reflections
4623 reflections with *I* > 2σ(*I*)
*R*_int_ = 0.042

#### Refinement

*R*[*F* ^2^ > 2σ(*F* ^2^)] = 0.029
*wR*(*F* ^2^) = 0.066
*S* = 1.06
5393 reflections
284 parameters
H-atom parameters constrained
Δρ_max_ = 0.90 e Å^−3^
Δρ_min_ = −0.51 e Å^−3^

### 

Data collection: *CrysAlis PRO* (Oxford Diffraction, 2010 ▶); cell refinement: *CrysAlis PRO*; data reduction: *CrysAlis PRO*; program(s) used to solve structure: *SHELXS97* (Sheldrick, 2008 ▶); program(s) used to refine structure: *SHELXL97* (Sheldrick, 2008 ▶); molecular graphics: *ORTEP-3* (Farrugia, 1997 ▶) and *DIAMOND* (Brandenburg, 2006 ▶); software used to prepare material for publication: *publCIF* (Westrip, 2010 ▶).

## Supplementary Material

Crystal structure: contains datablocks global, I. DOI: 10.1107/S1600536811006878/pk2304sup1.cif

Structure factors: contains datablocks I. DOI: 10.1107/S1600536811006878/pk2304Isup2.hkl

Additional supplementary materials:  crystallographic information; 3D view; checkCIF report

## Figures and Tables

**Table 1:** Selected bond lengths (Å)

Cd—S1	2.6104 (7)
Cd—S2	2.7685 (7)
Cd—S3	2.6468 (7)
Cd—S4	2.6783 (7)
Cd—N3	2.379 (2)
Cd—N4	2.441 (2)

**Table 2:** Hydrogen-bond geometry (Å, °)

*D*—H⋯*A*	*D*—H	H⋯*A*	*D*⋯*A*	*D*—H⋯*A*
C16—H16⋯S3^i^	0.95	2.74	3.685 (3)	172

Symmetry code: (i) 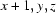.

**Fig. 1.**
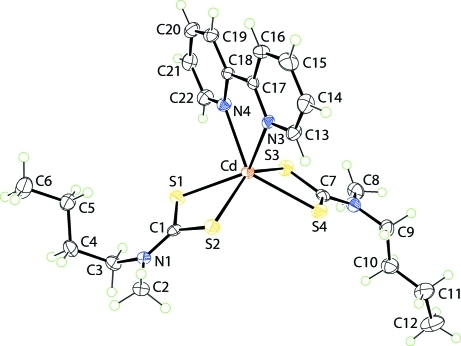
The molecular structure of (I) showing the atom-labelling scheme and displacement ellipsoids at the 50% probability level.

**Fig. 2.**
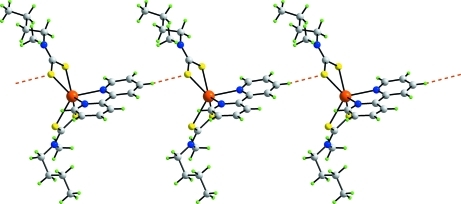
A view of the linear supramolecular chain along the a axis in (I) showing C—H···S contacts shown as orange dashed lines.

**Fig. 3.**
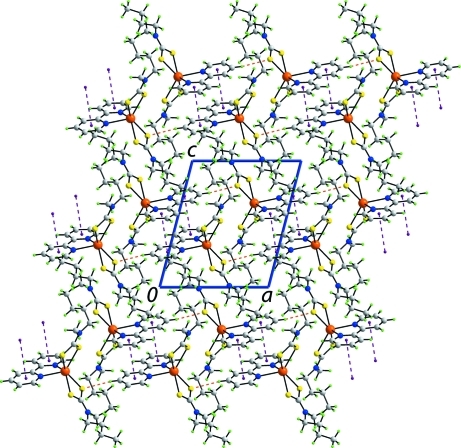
A view in projection down the b axis of the unit-cell contents for (I) showing supramolecular layers stacking along the c axis. The intermolecular C–H···S and π–π contacts are shown as orange and purple dashed lines, respectively.

## supplementary crystallographic information

### Comment

Adducts related to the title compound, (I), attract attention in crystal engineering studies (Chen *et al.*, 2006; Benson *et al.*, 2007). The Cd^II^ atom in (I) is six-coordinated, being chelated by two almost symmetrically coordinating dithiocarbamate ligands, and the N donor atoms of 2,2'-bipyridyl ligand, Fig. 1 and Table 1. The coordination geometry is intermediate between trigonal prismatic and octahedral with a leaning towards the former. The angle between the triangular faces defined by the S1,S3,N4 and S2,S4,N3 atoms is 5.36 (9) °, and these are twisted by approximately 13 ° about the axis through them, compared to 0 ° for an an ideal trigonal prism and 60 ° for an ideal octahedron. The symmetric mode of coordination of the dithiocarbamate ligands is reflected in the associated C≐S bond distances which lie in the narrow range of 1.721 (2) to 1.733 (3) Å. The mode of coordination of the dithiocarbamate ligand, the disposition of the ligand donor set, and the intermediate coordination geometry observed for (I) matches a literature precedent (Song & Tiekink, 2009).

Linear supramolecular chains along the *a* axis are formed in the crystal structure *via* C—H···S interactions, Table 2 and Fig. 2. These are consolidated into layers in the *ab* plane by π–π interactions formed between the pyridyl rings [*Cg*(N3,C14–C18)···*Cg*(N4,C19–C23)^i^ = 3.6587 (13) Å with angle between rings = 5.35 (11) ° for *i*: 2 - *x*, 1 - *y*, 1 - *z*]. Supramolecular layers stack along the *c* axis, Fig. 3.

### Experimental

The title compound was prepared using an *in situ* method. The first step was the addition of carbon disulfide (0.03 mol) to an ethanolic solution (20 ml) of butylmethylamine (0.03 mol) in ethanol (20 ml). The mixture was stirred for 1 h at 277 K. The resulting solution was added drop wise to a solution of cadmium(II) dichloride (0.015 mol) in ethanol (20 ml) followed by stirring for 4 h. A white precipitate was formed, filtered and washed with cold ethanol. The precipitate, Cd(C_6_H_12_NS_2_)_2_ (0.01 mol), and 2,2'-bipyridyl (0.01 mol) were dissolved together in chloroform (20 ml) and stirred for 1 h. A yellowish precipitate was formed, filtered and dried in a desiccator. Crystallization was from its ethanol:chloroform (1:2) solution. Yield 86%; *M*.pt. 424–426 K. Elemental analysis. Found (calculated) for C_22_H_32_CdN_4_S_4_: C, 44.21 (44.156); H 5.32 (5.40); Cd 18.54 (18.96); N 9.23 (9.40); S 21.45 (21.63) %. UV (CHCl_3_) λ_max_ 284 (*L*(π) →*L*(π*)). IR (KBr): ν(C—H) 2929*m*; ν(C≐N) 1485*m*; ν(N—C) 1158 s; ν(C≐S) 974 s; ν(Cd—S) 354 s cm^-1^.

### Refinement

Carbon-bound H-atoms were placed in calculated positions (C—H 0.95 to 0.99 Å) and were included in the refinement in the riding model approximation, with *U*_iso_(H) set to 1.2 to 1.5*U*_equiv_(C).

### Crystal data

**Table:**

[Cd(C_6_H_12_NS_2_)_2_(C_10_H_8_N_2_)]	*Z* = 2
*M**_r_* = 593.16	*F*(000) = 608
Triclinic, *P*1	*D*_x_ = 1.512 Mg m^−^^3^
Hall symbol: -P 1	Mo *K*α radiation, λ = 0.71073 Å
*a* = 10.3215 (4) Å	Cell parameters from 8976 reflections
*b* = 10.6465 (4) Å	θ = 2.0–29.0°
*c* = 12.4546 (5) Å	µ = 1.18 mm^−^^1^
α = 81.566 (3)°	*T* = 150 K
β = 74.790 (3)°	Plate, colourless
γ = 83.641 (3)°	0.27 × 0.16 × 0.01 mm
*V* = 1302.64 (9) Å^3^	

### Data collection

**Table:**

Oxford Diffraction Xcaliber Eos Gemini diffractometer	5393 independent reflections
Radiation source: fine-focus sealed tube	4623 reflections with *I* > 2σ(*I*)
graphite	*R*_int_ = 0.042
Detector resolution: 16.1952 pixels mm^-1^	θ_max_ = 26.5°, θ_min_ = 2.3°
ω scans	*h* = −12→12
Absorption correction: multi-scan (*CrysAlis PRO*; Oxford Diffraction, 2010)	*k* = −13→13
*T*_min_ = 0.829, *T*_max_ = 0.990	*l* = −15→15
15837 measured reflections	

### Refinement

**Table:**

Refinement on *F*^2^	Primary atom site location: structure-invariant direct methods
Least-squares matrix: full	Secondary atom site location: difference Fourier map
*R*[*F*^2^ > 2σ(*F*^2^)] = 0.029	Hydrogen site location: inferred from neighbouring sites
*wR*(*F*^2^) = 0.066	H-atom parameters constrained
*S* = 1.06	*w* = 1/[σ^2^(*F*_o_^2^) + (0.0248*P*)^2^ + 0.0565*P*] where *P* = (*F*_o_^2^ + 2*F*_c_^2^)/3
5393 reflections	(Δ/σ)_max_ = 0.001
284 parameters	Δρ_max_ = 0.90 e Å^−^^3^
0 restraints	Δρ_min_ = −0.51 e Å^−^^3^

### Special details

**Table:**

Geometry. All s.u.'s (except the s.u. in the dihedral angle between two l.s. planes) are estimated using the full covariance matrix. The cell s.u.'s are taken into account individually in the estimation of s.u.'s in distances, angles and torsion angles; correlations between s.u.'s in cell parameters are only used when they are defined by crystal symmetry. An approximate (isotropic) treatment of cell s.u.'s is used for estimating s.u.'s involving l.s. planes.
Refinement. Refinement of *F*^2^ against ALL reflections. The weighted *R*-factor *wR* and goodness of fit *S* are based on *F*^2^, conventional *R*-factors *R* are based on *F*, with *F* set to zero for negative *F*^2^. The threshold expression of *F*^2^ > 2σ(*F*^2^) is used only for calculating *R*-factors(gt) *etc*. and is not relevant to the choice of reflections for refinement. *R*-factors based on *F*^2^ are statistically about twice as large as those based on *F*, and *R*- factors based on ALL data will be even larger.

### Fractional atomic coordinates and isotropic or equivalent isotropic displacement parameters (Å^2^)

**Table:**

	*x*	*y*	*z*	*U*_iso_*/*U*_eq_	
Cd	0.669262 (17)	0.668974 (17)	0.664886 (14)	0.02060 (7)	
S1	0.60096 (7)	0.69901 (6)	0.47470 (5)	0.02501 (15)	
S2	0.73388 (7)	0.90040 (6)	0.53865 (5)	0.02474 (15)	
S3	0.47202 (7)	0.54259 (7)	0.80306 (6)	0.02816 (16)	
S4	0.56208 (7)	0.77897 (6)	0.85175 (5)	0.02633 (15)	
N1	0.6294 (2)	0.93184 (19)	0.36194 (16)	0.0217 (5)	
N2	0.3637 (2)	0.6493 (2)	0.98885 (17)	0.0274 (5)	
N3	0.8838 (2)	0.62383 (19)	0.70520 (15)	0.0188 (4)	
N4	0.7756 (2)	0.46118 (19)	0.61699 (16)	0.0220 (5)	
C1	0.6525 (2)	0.8525 (2)	0.44916 (19)	0.0208 (5)	
C2	0.6688 (3)	1.0633 (2)	0.3422 (2)	0.0274 (6)	
H2A	0.6156	1.1102	0.4032	0.041*	
H2B	0.6525	1.1048	0.2708	0.041*	
H2C	0.7647	1.0626	0.3393	0.041*	
C3	0.5565 (3)	0.9003 (2)	0.2837 (2)	0.0261 (6)	
H3A	0.4858	0.9693	0.2754	0.031*	
H3B	0.5114	0.8209	0.3156	0.031*	
C4	0.6490 (3)	0.8829 (3)	0.1684 (2)	0.0286 (6)	
H4A	0.5930	0.8765	0.1162	0.034*	
H4B	0.7002	0.9593	0.1401	0.034*	
C5	0.7477 (3)	0.7662 (3)	0.1679 (2)	0.0300 (6)	
H5A	0.6968	0.6893	0.1940	0.036*	
H5B	0.8026	0.7713	0.2212	0.036*	
C6	0.8409 (3)	0.7531 (3)	0.0524 (2)	0.0453 (8)	
H6A	0.7872	0.7466	−0.0006	0.068*	
H6B	0.9021	0.6764	0.0565	0.068*	
H6C	0.8933	0.8280	0.0269	0.068*	
C7	0.4562 (2)	0.6567 (2)	0.8919 (2)	0.0232 (6)	
C8	0.2758 (3)	0.5432 (3)	1.0244 (2)	0.0350 (7)	
H8A	0.2020	0.5592	0.9870	0.053*	
H8B	0.2387	0.5358	1.1058	0.053*	
H8C	0.3281	0.4638	1.0041	0.053*	
C9	0.3423 (3)	0.7429 (3)	1.0687 (2)	0.0329 (7)	
H9A	0.4118	0.8051	1.0410	0.039*	
H9B	0.3536	0.6986	1.1417	0.039*	
C11	0.2043 (3)	0.8137 (3)	1.0860 (2)	0.0350 (7)	
H11A	0.1347	0.7521	1.1166	0.042*	
H11B	0.1916	0.8560	1.0128	0.042*	
C12	0.1862 (3)	0.9133 (3)	1.1660 (2)	0.0400 (7)	
H12A	0.2177	0.8748	1.2328	0.048*	
H12B	0.2427	0.9843	1.1285	0.048*	
C13	0.0408 (3)	0.9657 (3)	1.2032 (3)	0.0484 (9)	
H13A	0.0119	1.0120	1.1383	0.073*	
H13B	0.0329	1.0237	1.2592	0.073*	
H13C	−0.0165	0.8953	1.2361	0.073*	
C14	0.9307 (3)	0.7054 (2)	0.7554 (2)	0.0249 (6)	
H14	0.8736	0.7775	0.7797	0.030*	
C15	1.0578 (3)	0.6896 (3)	0.7734 (2)	0.0299 (6)	
H15	1.0872	0.7486	0.8106	0.036*	
C16	1.1413 (3)	0.5869 (3)	0.7364 (2)	0.0327 (7)	
H16	1.2303	0.5749	0.7459	0.039*	
C17	1.0942 (3)	0.5005 (2)	0.6848 (2)	0.0257 (6)	
H17	1.1504	0.4285	0.6590	0.031*	
C18	0.9638 (2)	0.5211 (2)	0.67166 (18)	0.0185 (5)	
C19	0.9038 (2)	0.4298 (2)	0.62209 (19)	0.0190 (5)	
C20	0.9733 (3)	0.3185 (2)	0.5849 (2)	0.0243 (6)	
H20	1.0649	0.2995	0.5867	0.029*	
C21	0.9076 (3)	0.2359 (2)	0.5453 (2)	0.0299 (6)	
H21	0.9535	0.1592	0.5200	0.036*	
C22	0.7746 (3)	0.2662 (3)	0.5428 (2)	0.0304 (6)	
H22	0.7265	0.2101	0.5177	0.037*	
C23	0.7136 (3)	0.3805 (3)	0.5781 (2)	0.0287 (6)	
H23	0.6231	0.4030	0.5744	0.034*	

### Atomic displacement parameters (Å^2^)

**Table:**

	*U*^11^	*U*^22^	*U*^33^	*U*^12^	*U*^13^	*U*^23^
Cd	0.01478 (10)	0.02502 (11)	0.02106 (10)	0.00349 (7)	−0.00428 (8)	−0.00391 (7)
S1	0.0271 (4)	0.0231 (3)	0.0261 (3)	−0.0007 (3)	−0.0104 (3)	−0.0012 (3)
S2	0.0229 (3)	0.0269 (4)	0.0250 (3)	0.0008 (3)	−0.0077 (3)	−0.0042 (3)
S3	0.0179 (3)	0.0379 (4)	0.0285 (4)	−0.0019 (3)	−0.0040 (3)	−0.0068 (3)
S4	0.0222 (3)	0.0264 (4)	0.0254 (3)	0.0025 (3)	0.0001 (3)	−0.0016 (3)
N1	0.0219 (12)	0.0217 (11)	0.0210 (11)	0.0041 (9)	−0.0064 (9)	−0.0031 (9)
N2	0.0221 (12)	0.0335 (13)	0.0215 (11)	−0.0001 (10)	−0.0003 (10)	0.0018 (9)
N3	0.0158 (11)	0.0228 (11)	0.0161 (10)	−0.0001 (9)	−0.0010 (9)	−0.0037 (8)
N4	0.0172 (11)	0.0250 (12)	0.0240 (11)	−0.0005 (9)	−0.0045 (9)	−0.0050 (9)
C1	0.0137 (12)	0.0230 (13)	0.0221 (13)	0.0065 (10)	0.0000 (10)	−0.0050 (10)
C2	0.0314 (15)	0.0216 (14)	0.0284 (14)	0.0027 (11)	−0.0085 (12)	−0.0022 (11)
C3	0.0248 (14)	0.0284 (15)	0.0270 (14)	0.0011 (12)	−0.0123 (12)	−0.0009 (11)
C4	0.0344 (16)	0.0305 (15)	0.0232 (13)	−0.0031 (12)	−0.0128 (13)	0.0002 (11)
C5	0.0317 (16)	0.0310 (15)	0.0271 (14)	−0.0011 (12)	−0.0055 (13)	−0.0069 (12)
C6	0.048 (2)	0.054 (2)	0.0311 (16)	0.0015 (16)	−0.0035 (15)	−0.0122 (14)
C7	0.0152 (13)	0.0302 (14)	0.0215 (13)	0.0075 (11)	−0.0060 (11)	0.0014 (11)
C8	0.0290 (16)	0.0405 (17)	0.0288 (15)	−0.0032 (13)	−0.0003 (13)	0.0053 (12)
C9	0.0273 (15)	0.0460 (18)	0.0215 (13)	0.0018 (13)	−0.0010 (12)	−0.0041 (12)
C11	0.0304 (16)	0.0350 (16)	0.0338 (16)	0.0017 (13)	−0.0018 (13)	−0.0003 (13)
C12	0.0358 (18)	0.0343 (17)	0.0392 (17)	−0.0016 (14)	0.0066 (15)	−0.0009 (13)
C13	0.0395 (19)	0.0323 (18)	0.061 (2)	0.0016 (15)	0.0080 (17)	−0.0069 (15)
C14	0.0211 (14)	0.0278 (14)	0.0253 (13)	0.0004 (11)	−0.0039 (11)	−0.0073 (11)
C15	0.0240 (15)	0.0383 (17)	0.0312 (15)	−0.0058 (13)	−0.0098 (13)	−0.0086 (12)
C16	0.0184 (14)	0.0440 (18)	0.0383 (16)	−0.0010 (13)	−0.0133 (13)	−0.0032 (13)
C17	0.0200 (14)	0.0277 (14)	0.0282 (14)	0.0059 (11)	−0.0071 (12)	−0.0036 (11)
C18	0.0153 (12)	0.0236 (13)	0.0136 (11)	−0.0004 (10)	−0.0012 (10)	0.0022 (10)
C19	0.0173 (13)	0.0191 (13)	0.0172 (12)	0.0008 (10)	−0.0007 (10)	0.0005 (9)
C20	0.0189 (13)	0.0248 (14)	0.0242 (13)	0.0002 (11)	0.0009 (11)	0.0003 (11)
C21	0.0354 (16)	0.0219 (14)	0.0283 (14)	−0.0002 (12)	−0.0004 (13)	−0.0054 (11)
C22	0.0337 (16)	0.0295 (15)	0.0290 (14)	−0.0090 (13)	−0.0048 (13)	−0.0064 (12)
C23	0.0217 (14)	0.0325 (16)	0.0325 (15)	−0.0020 (12)	−0.0057 (12)	−0.0075 (12)

### Geometric parameters (Å, °)

**Table:**

Cd—S1	2.6104 (7)	C6—H6C	0.9800
Cd—S2	2.7685 (7)	C8—H8A	0.9800
Cd—S3	2.6468 (7)	C8—H8B	0.9800
Cd—S4	2.6783 (7)	C8—H8C	0.9800
Cd—N3	2.379 (2)	C9—C11	1.514 (4)
Cd—N4	2.441 (2)	C9—H9A	0.9900
S1—C1	1.733 (3)	C9—H9B	0.9900
S2—C1	1.721 (2)	C11—C12	1.522 (4)
S3—C7	1.727 (3)	C11—H11A	0.9900
S4—C7	1.725 (3)	C11—H11B	0.9900
N1—C1	1.332 (3)	C12—C13	1.518 (4)
N1—C2	1.469 (3)	C12—H12A	0.9900
N1—C3	1.471 (3)	C12—H12B	0.9900
N2—C7	1.327 (3)	C13—H13A	0.9800
N2—C9	1.469 (3)	C13—H13B	0.9800
N2—C8	1.472 (3)	C13—H13C	0.9800
N3—C14	1.338 (3)	C14—C15	1.377 (4)
N3—C18	1.344 (3)	C14—H14	0.9500
N4—C23	1.337 (3)	C15—C16	1.372 (4)
N4—C19	1.344 (3)	C15—H15	0.9500
C2—H2A	0.9800	C16—C17	1.389 (4)
C2—H2B	0.9800	C16—H16	0.9500
C2—H2C	0.9800	C17—C18	1.388 (3)
C3—C4	1.526 (3)	C17—H17	0.9500
C3—H3A	0.9900	C18—C19	1.489 (3)
C3—H3B	0.9900	C19—C20	1.390 (3)
C4—C5	1.516 (4)	C20—C21	1.382 (4)
C4—H4A	0.9900	C20—H20	0.9500
C4—H4B	0.9900	C21—C22	1.383 (4)
C5—C6	1.522 (4)	C21—H21	0.9500
C5—H5A	0.9900	C22—C23	1.383 (4)
C5—H5B	0.9900	C22—H22	0.9500
C6—H6A	0.9800	C23—H23	0.9500
C6—H6B	0.9800		
			
N3—Cd—N4	67.00 (7)	N2—C7—S4	121.3 (2)
N3—Cd—S1	130.89 (5)	N2—C7—S3	119.9 (2)
N4—Cd—S1	87.19 (5)	S4—C7—S3	118.79 (14)
N3—Cd—S3	115.46 (5)	N2—C8—H8A	109.5
N4—Cd—S3	86.24 (5)	N2—C8—H8B	109.5
S1—Cd—S3	102.91 (2)	H8A—C8—H8B	109.5
N3—Cd—S4	93.32 (5)	N2—C8—H8C	109.5
N4—Cd—S4	137.16 (5)	H8A—C8—H8C	109.5
S1—Cd—S4	130.38 (2)	H8B—C8—H8C	109.5
S3—Cd—S4	67.82 (2)	N2—C9—C11	112.8 (2)
N3—Cd—S2	94.02 (5)	N2—C9—H9A	109.0
N4—Cd—S2	125.32 (5)	C11—C9—H9A	109.0
S1—Cd—S2	67.03 (2)	N2—C9—H9B	109.0
S3—Cd—S2	144.43 (2)	C11—C9—H9B	109.0
S4—Cd—S2	92.26 (2)	H9A—C9—H9B	107.8
C1—S1—Cd	89.21 (8)	C9—C11—C12	111.9 (3)
C1—S2—Cd	84.39 (8)	C9—C11—H11A	109.2
C7—S3—Cd	87.18 (9)	C12—C11—H11A	109.2
C7—S4—Cd	86.21 (9)	C9—C11—H11B	109.2
C1—N1—C2	120.7 (2)	C12—C11—H11B	109.2
C1—N1—C3	124.2 (2)	H11A—C11—H11B	107.9
C2—N1—C3	114.95 (19)	C13—C12—C11	112.5 (3)
C7—N2—C9	123.5 (2)	C13—C12—H12A	109.1
C7—N2—C8	121.3 (2)	C11—C12—H12A	109.1
C9—N2—C8	115.2 (2)	C13—C12—H12B	109.1
C14—N3—C18	118.6 (2)	C11—C12—H12B	109.1
C14—N3—Cd	120.22 (16)	H12A—C12—H12B	107.8
C18—N3—Cd	121.00 (15)	C12—C13—H13A	109.5
C23—N4—C19	118.4 (2)	C12—C13—H13B	109.5
C23—N4—Cd	122.04 (16)	H13A—C13—H13B	109.5
C19—N4—Cd	119.35 (15)	C12—C13—H13C	109.5
N1—C1—S2	120.62 (19)	H13A—C13—H13C	109.5
N1—C1—S1	120.58 (19)	H13B—C13—H13C	109.5
S2—C1—S1	118.80 (14)	N3—C14—C15	123.0 (2)
N1—C2—H2A	109.5	N3—C14—H14	118.5
N1—C2—H2B	109.5	C15—C14—H14	118.5
H2A—C2—H2B	109.5	C16—C15—C14	118.6 (2)
N1—C2—H2C	109.5	C16—C15—H15	120.7
H2A—C2—H2C	109.5	C14—C15—H15	120.7
H2B—C2—H2C	109.5	C15—C16—C17	119.3 (2)
N1—C3—C4	112.5 (2)	C15—C16—H16	120.3
N1—C3—H3A	109.1	C17—C16—H16	120.3
C4—C3—H3A	109.1	C18—C17—C16	118.9 (2)
N1—C3—H3B	109.1	C18—C17—H17	120.6
C4—C3—H3B	109.1	C16—C17—H17	120.6
H3A—C3—H3B	107.8	N3—C18—C17	121.5 (2)
C5—C4—C3	113.9 (2)	N3—C18—C19	116.3 (2)
C5—C4—H4A	108.8	C17—C18—C19	122.1 (2)
C3—C4—H4A	108.8	N4—C19—C20	121.6 (2)
C5—C4—H4B	108.8	N4—C19—C18	115.3 (2)
C3—C4—H4B	108.8	C20—C19—C18	123.1 (2)
H4A—C4—H4B	107.7	C21—C20—C19	119.2 (2)
C4—C5—C6	112.7 (2)	C21—C20—H20	120.4
C4—C5—H5A	109.1	C19—C20—H20	120.4
C6—C5—H5A	109.1	C20—C21—C22	119.3 (2)
C4—C5—H5B	109.1	C20—C21—H21	120.3
C6—C5—H5B	109.1	C22—C21—H21	120.3
H5A—C5—H5B	107.8	C21—C22—C23	118.0 (2)
C5—C6—H6A	109.5	C21—C22—H22	121.0
C5—C6—H6B	109.5	C23—C22—H22	121.0
H6A—C6—H6B	109.5	N4—C23—C22	123.4 (2)
C5—C6—H6C	109.5	N4—C23—H23	118.3
H6A—C6—H6C	109.5	C22—C23—H23	118.3
H6B—C6—H6C	109.5		
			
N3—Cd—S1—C1	−78.98 (10)	Cd—S2—C1—S1	−7.09 (13)
N4—Cd—S1—C1	−134.92 (9)	Cd—S1—C1—N1	−172.43 (19)
S3—Cd—S1—C1	139.60 (8)	Cd—S1—C1—S2	7.49 (13)
S4—Cd—S1—C1	67.87 (8)	C1—N1—C3—C4	−108.9 (3)
S2—Cd—S1—C1	−4.42 (8)	C2—N1—C3—C4	75.2 (3)
N3—Cd—S2—C1	137.55 (9)	N1—C3—C4—C5	68.0 (3)
N4—Cd—S2—C1	73.04 (10)	C3—C4—C5—C6	−178.6 (2)
S1—Cd—S2—C1	4.47 (8)	C9—N2—C7—S4	0.6 (3)
S3—Cd—S2—C1	−75.42 (9)	C8—N2—C7—S4	−178.37 (19)
S4—Cd—S2—C1	−128.96 (8)	C9—N2—C7—S3	−179.28 (19)
N3—Cd—S3—C7	82.82 (10)	C8—N2—C7—S3	1.8 (3)
N4—Cd—S3—C7	145.11 (9)	Cd—S4—C7—N2	−179.9 (2)
S1—Cd—S3—C7	−128.65 (8)	Cd—S4—C7—S3	−0.02 (13)
S4—Cd—S3—C7	−0.01 (8)	Cd—S3—C7—N2	179.86 (19)
S2—Cd—S3—C7	−60.22 (9)	Cd—S3—C7—S4	0.02 (13)
N3—Cd—S4—C7	−116.17 (9)	C7—N2—C9—C11	116.6 (3)
N4—Cd—S4—C7	−57.04 (11)	C8—N2—C9—C11	−64.4 (3)
S1—Cd—S4—C7	88.29 (8)	N2—C9—C11—C12	−178.0 (2)
S3—Cd—S4—C7	0.01 (8)	C9—C11—C12—C13	−168.1 (3)
S2—Cd—S4—C7	149.67 (8)	C18—N3—C14—C15	0.6 (4)
N4—Cd—N3—C14	−176.21 (19)	Cd—N3—C14—C15	−174.99 (19)
S1—Cd—N3—C14	119.77 (16)	N3—C14—C15—C16	1.2 (4)
S3—Cd—N3—C14	−102.54 (17)	C14—C15—C16—C17	−1.7 (4)
S4—Cd—N3—C14	−35.56 (17)	C15—C16—C17—C18	0.3 (4)
S2—Cd—N3—C14	56.94 (17)	C14—N3—C18—C17	−2.1 (3)
N4—Cd—N3—C18	8.28 (16)	Cd—N3—C18—C17	173.52 (17)
S1—Cd—N3—C18	−55.74 (19)	C14—N3—C18—C19	176.7 (2)
S3—Cd—N3—C18	81.95 (17)	Cd—N3—C18—C19	−7.7 (3)
S4—Cd—N3—C18	148.92 (16)	C16—C17—C18—N3	1.6 (4)
S2—Cd—N3—C18	−118.57 (16)	C16—C17—C18—C19	−177.1 (2)
N3—Cd—N4—C23	176.6 (2)	C23—N4—C19—C20	2.0 (3)
S1—Cd—N4—C23	−46.31 (18)	Cd—N4—C19—C20	−173.34 (17)
S3—Cd—N4—C23	56.83 (18)	C23—N4—C19—C18	−177.0 (2)
S4—Cd—N4—C23	107.98 (18)	Cd—N4—C19—C18	7.6 (3)
S2—Cd—N4—C23	−105.41 (18)	N3—C18—C19—N4	−0.2 (3)
N3—Cd—N4—C19	−8.27 (16)	C17—C18—C19—N4	178.6 (2)
S1—Cd—N4—C19	128.86 (17)	N3—C18—C19—C20	−179.2 (2)
S3—Cd—N4—C19	−128.00 (17)	C17—C18—C19—C20	−0.4 (4)
S4—Cd—N4—C19	−76.85 (19)	N4—C19—C20—C21	−2.3 (4)
S2—Cd—N4—C19	69.76 (18)	C18—C19—C20—C21	176.7 (2)
C2—N1—C1—S2	−2.0 (3)	C19—C20—C21—C22	0.4 (4)
C3—N1—C1—S2	−177.74 (18)	C20—C21—C22—C23	1.5 (4)
C2—N1—C1—S1	177.93 (18)	C19—N4—C23—C22	0.1 (4)
C3—N1—C1—S1	2.2 (3)	Cd—N4—C23—C22	175.32 (19)
Cd—S2—C1—N1	172.83 (19)	C21—C22—C23—N4	−1.9 (4)

### Hydrogen-bond geometry (Å, °)

**Table:**

*D*—H···*A*	*D*—H	H···*A*	*D*···*A*	*D*—H···*A*
C16—H16···S3^i^	0.95	2.74	3.685 (3)	172

Symmetry codes: (i) *x*+1, *y*, *z*.
